# An Empirical Evaluation of Low-Rank Adapted Vision–Language Models for Radiology Image Captioning

**DOI:** 10.3390/bioengineering12121330

**Published:** 2025-12-05

**Authors:** Mahmudul Hoque, Raisa Nusrat Chowdhury, Md Rakibul Hasan, Ojonugwa Oluwafemi Ejiga Peter, Fahmi Khalifa, Md Mahmudur Rahman

**Affiliations:** 1 Department of Computer Science, Morgan State University, Baltimore, MD 21251, USA; 2 Department of Information Systems and Cybersecurity, The University of Texas at San Antonio, San Antonio, TX 78249, USA; 3 Department of Electrical and Computer Engineering, Morgan State University, Baltimore, MD 21251, USA

**Keywords:** vision-language models, medical image captioning, radiology report generation, Low-Rank Adaptation, ROCOv2 dataset, caption quality, parameter-efficient fine-tuning

## Abstract

Rapidly growing medical imaging volumes have increased radiologist workloads, creating demand for automated tools that support interpretation and reduce reporting delays. Vision-language models (VLMs) can generate clinically relevant captions to accelerate report drafting, yet their varying parameter scales require systematic evaluation for clinical utility. This study evaluated ten multimodal models fine-tuned on the Radiology Objects in Context version 2 (ROCOv2) dataset containing 116,635 images across eight modalities. We compared four Large VLMs (LVLMs) including LLaVA variants and IDEFICS-9B against four Small VLMs (SVLMs) including MoonDream2, Qwen variants, and SmolVLM, alongside two fully fine-tuned baseline architectures (VisionGPT2 and CNN-Transformer). Low-Rank Adaptation (LoRA), applied to fewer than 1% of selected model parameters, proved optimal among adaptation strategies, outperforming broader LoRA configurations. Models were assessed on relevance (semantic similarity) and factuality (concept-level correctness) metrics. Performance showed clear stratification: LVLMs (0.273 to 0.317 overall), SVLMs (0.188 to 0.279), and baselines (0.154 to 0.177). LLaVA-Mistral-7B achieved the highest performance with relevance and factuality scores of 0.516 and 0.118, respectively, substantially exceeding the VisionGPT2 baseline (0.325, 0.028). Among the SVLMs, MoonDream2 demonstrated competitive relevance (0.466), approaching the performance of some LVLMs despite its smaller size. To investigate performance enhancement strategies for underperforming SVLMs, we prepended predicted imaging modality labels at inference time, which yielded variable results. These findings provide quantitative benchmarks for VLM selection in medical imaging, demonstrating that while model scale influences performance, architectural design and targeted adaptation enable select compact models to achieve competitive results.

## 1. Introduction

Radiology occupies a central role in contemporary healthcare, serving as a fundamental tool in the diagnosis, treatment planning, and monitoring of a myriad of diseases [1]. Despite its importance, the field is facing an imbalance between the demand for imaging diagnosis and the supply of radiologists. The number of diagnostic studies has exploded exponentially over the past decade, while the radiologist workforce remains nearly stagnant, resulting in substantially increased workload per radiologist [2]. Furthermore, technological advances in medical imaging have led to examinations that generate hundreds of images per study, compounding the interpretation burden beyond what simple case numbers suggest [2]. Such heavy caseloads can translate into significant delays in report turnaround times, with overburdened specialists struggling to keep pace with ever-expanding worklists [3]. Moreover, interpretation variability is an inherent challenge as different radiologists may provide differing descriptions or even miss findings under stress, leading to inconsistencies in diagnostic reports [3]. These workflow bottlenecks and potential discrepancies in reporting highlight a pressing need for automated tools that can generate preliminary image captions or draft reports, helping to reduce turnaround time and standardize diagnostic descriptions across practitioners [3]. In this context, automated radiology image captioning has emerged as an important research direction, aiming to have artificial intelligence (AI) systems produce clinically relevant descriptions of medical images that can assist radiologists and mitigate delays in care [4].

Recent advances in AI offer promising avenues to address these challenges. In particular, VLMs have shown remarkable capabilities in translating images into text by combining visual processing with natural language generation [4]. By integrating vision and language modalities, VLMs achieve a more holistic understanding of complex data and can perform sophisticated image captioning task. General-purpose VLMs like Large Language and Vision Assistants (LLaVA) [5], DeepMind’s Flamingo [6], and Contrastive Language–Image Pre-training (CLIP) [7] have demonstrated high performance on broad image–text benchmarks. Building on these successes, specialized medical VLMs are now emerging. For example, BioViL [8], Med-Flamingo [9], and RadFM [10] adapt multimodal pretraining to clinical data and have been shown to better capture the nuanced visual–textual patterns of medical imagery. Using knowledge from large-scale multimodal pre-training, VLMs can assist in detecting subtle abnormalities on imaging that might be difficult for the human eye to spot [11]. The appeal of VLM-based captioning in radiology is thus twofold: it could expedite the reporting process by automatically generating draft descriptions of findings, and it could provide more consistent, standardized captions that reduce subjective variability [12].

Importantly, VLMs today span a wide spectrum of model sizes and complexities [11]. On one end are large-scale VLMs with many billions of parameters (for instance, while the original IDEFICS model contains 80 B parameters and represents the upper extreme, more practical variants like IDEFICS-9B offer a balance of capability and deployability) [12]. These larger models often achieve state-of-the-art (SOTA) accuracy and can produce very detailed, fluent image descriptions. However, their size comes with substantial drawbacks. They require enormous computing resources for training and inference, typically needing multiple high-memory GPUs or specialized hardware accelerators. Integrating such a model into a clinical pipeline can be computationally prohibitive, as these systems tend to be resource-intensive and difficult to deploy on standard workstations. On the other end are lightweight VLMs with on the order of a few hundred million to a few billion parameters [13]. These compact models are designed for efficiency and easier deployment. They can often run on a single-commodity GPU, making them attractive for smaller hospitals or research labs without access to extensive computing infrastructure. The trade-off, however, is that smaller models may exhibit lower raw performance. They may miss finer details or produce less fluent captions compared to their larger counterparts. This dichotomy between large and small VLMs raises an important challenge for the field, namely the need to balance model accuracy and descriptive detail with considerations of computational efficiency and resource availability [14]. Addressing this trade-off represents an important research direction toward eventual integration of AI-based image captioning systems into clinical workflows.

A key limitation of the LVLMs is the difficulty of fine-tuning and deploying them in typical clinical research settings [14]. Fully fine-tuning a multi-billion-parameter model for a specific task (such as radiology captioning) is computationally expensive and memory-intensive, often to the point of impracticality [15]. For many clinical settings, it is not feasible to dedicate the necessary GPU clusters or prolonged training time required for traditional fine-tuning of a giant model on local data [16]. Furthermore, even after training, inference with an LVLM can be slow and require significant GPU memory, impeding real-time use [17]. These factors have motivated a shift toward methods that favor deployability on standard hardware over brute-force model training [17]. In practice, it is often more desirable to take a strong pre-trained VLM and adapt it in a lightweight manner that can run on an everyday workstation, rather than to re-train or heavily fine-tune it, which requires specialized hardware [18].

In response, researchers have developed parameter-efficient fine-tuning (PEFT) techniques that significantly reduce the resources needed to adapt large models [16]. PEFT approaches adjust only a small fraction of the model’s parameters while keeping most weights of the pre-trained model frozen. This drastically lowers the computational and memory overhead of training, making it possible to fine-tune large models on modest hardware [18]. Among these methods, Low-Rank Adaptation (LoRA) [19] has gained particular prominence for vision–language applications. LoRA inserts a pair of low-rank trainable weight matrices into each layer of the model, which are learned during fine-tuning, instead of modifying the full weight matrices [20]. This approach effectively injects task-specific knowledge into the model with only a few million additional parameters, all while preserving the original model’s weights. Our experiments demonstrate that PEFT with LoRA can surpass fully fine-tuned traditional architectures, validating the effectiveness of this approach. The growing use of PEFT means that even LVLMs containing tens of billions of parameters can be adapted to niche tasks like medical image captioning using a standard GPU without needing to retrain the entire network from scratch [16]. These advances in parameter-efficient adaptation represent progress toward the long-term goal of making advanced multimodal AI more accessible in clinical settings.

Despite rapid progress in VLM development, there are gaps in understanding how model scale and tuning strategies impact performance in medical image captioning [21]. First, there is a lack of unified, head-to-head comparisons of different sized models on the same clinical imaging dataset [4]. Much of the prior work evaluates models in isolation or on differing data, which makes it difficult to objectively determine how a 500-million-parameter model stacks up against a 9-billion-parameter model under similar conditions [20]. As a result, the field has had an unclear picture of the true trade-offs between clinical utility (caption quality) across the spectrum of model sizes. Second, existing image captioning studies in radiology have rarely assessed how well models generalize across multiple imaging modalities. Many works focus on a single modality (e.g., chest X-rays); far fewer have tested models on diverse modalities (e.g., X-rays, MRI, CT, etc.) to ensure the captioning approach is broadly applicable [4]. Indeed, models trained on one modality often struggle to interpret others without additional training. This modality-specific focus leaves open the question of how a given model’s performance might change when confronted with different image types or anatomies, an important consideration for real-world deployment. Third, to our knowledge, the published literature lacks a systematic comparison of parameter-efficient adaptation strategies for VLMs that empirically identifies optimal configurations for radiology captioning performance [5]. Additionally, inference-time augmentations, which are simple strategies applied at the time of caption generation to guide the model, have not been thoroughly investigated in this domain. For example, explicitly telling a model the modality of the image such as prepending the phrase “Radiograph:” or “CT:” to the input could, in theory, provide helpful context to a VLM [11]. Intuitively, this modality-aware prompting might remind the low-performing SVLMs of relevant domain knowledge and improve caption accuracy. Our investigation explores whether such interventions can compensate for reduced model capacity, potentially enabling smaller models to achieve competitive performance.

To provide additional context, we also benchmark these parameter-efficient fine-tuned VLMs against two baseline image captioning architectures from prior research [22]. The first is a fully fine-tuned CNN–Transformer model that uses a CNN network encoder similar to CheXNet paired with a Transformer-based decoder for language. This approach reflects traditional image captioning pipelines developed before the emergence of large multimodal models. The second is a fully fine-tuned custom VisionGPT2 model that employs a pre-trained GPT-2 language model conditioned on visual features–previously compared against a LoRA-adapted LLaVA-1.6-Mistral-7B in our preliminary investigation [14]. Including these baselines allows us to directly assess the performance gains of recent VLMs relative to earlier techniques.

We also investigate the effectiveness of modality-aware prompting as an inference-time intervention for the underperforming SVLMs [11]. In these experiments, the model is prepended with a text prompt indicating the image type (for instance, supplying “MRI:” or “ultrasound:” before the image is processed) to provide additional context. Our results show that this leads to only minor improvements in the fluency of captions generated by a few SVLMs, and does not meaningfully close the performance gap relative to the larger models. In other words, while adding modality cues can make a caption slightly more coherent or tailored for some models, it is not a substitute for the richer internal knowledge that LVLMs bring. The larger models consistently produce more detailed and clinically accurate captions, highlighting that model scale (and the breadth of training data it entails) remains a dominant factor in captioning quality.

This work extends our preliminary investigation [14], which evaluated LLaVA-1.6-Mistral-7B against a VisionGPT2 baseline for radiology image captioning. The previous study demonstrated that VLM adapted with LoRA could achieve competitive performance. However, it lacked a comparison of adaptation strategies, evaluation of alternative VLM architectures across different parameter scales, and structured assessment of caption quality dimensions. The current manuscript provides substantial advances across four key dimensions.

First, we introduce an ablation study to compare three adaptation strategies for LLaVA-1.6-Mistral-7B (targeted, extended, and hybrid spanning 40.1 M to 350.6 M parameters) and two strategies for LLaVA-1.6-Vicuna-7B, demonstrating that targeted adaptation using fewer parameters achieves superior performance. Second, we expand from two architectures to ten, incorporating LLaVA-1.6-Vicuna-7B, LLaVA-1.5-LLaMA-7B, and IDEFICS-9B, and four SVLMs (MoonDream2, Qwen 2-VL, Qwen-2.5, SmolVLM), plus CNN-Transformer baseline, enabling analysis across a wide range of model sizes. Third, we explore modality-aware prompting through ResNet-50 classification at inference time. Fourth, models were evaluated using a structured framework with composite metrics (Relevance Score and Factuality Score) that separate linguistic quality from clinical accuracy, incorporating existing metrics (UMLS Concepts F1 and AlignScore) not utilized in the conference version. Beyond these contributions, this manuscript includes parameter efficiency analysis, test case studies, and relevance–factuality correlation investigation. Table 1 summarizes these extensions.

Overall, this study provides a comparative benchmark of VLMs fine-tuned with LoRA for radiology captioning. By systematically evaluating models across scales from LVLMs to SVLMs, and reporting both parameter counts and adaptation targets, we clarify how model size and tuning strategies affect performance. In doing so, our work addresses gaps in prior studies by offering a comparison across model scales and establishing a reference point for future research. The following sections outline our methodology, present the experimental results, and discuss their implications for advancing medical image captioning.

## 2. Methodological Approach

The methodological framework of this study comprises four consecutive phases: data preparation, model preparation, training environment preparation, and post-training evaluation methods. Further discussions on this section, given below, will demonstrate the corresponding details of each phase.

### 2.1. Data Preparation

This study employs the Radiology Objects in Context version 2 (ROCOv2) dataset [23], comprising 116,635 radiological images from PubMed Central publications. The dataset is partitioned into training (80,091 images), validation (17,277 images), and test (19,267 images) sets, with each image paired with a caption and Unified Medical Language System (UMLS) concept annotations. UMLS provides standardized medical terminology through Concept Unique Identifiers (CUIs) [24]. This standardization enables consistent semantic representation of medical concepts across different vocabularies. Captions typically describe the imaging modality, anatomical region, and relevant findings, with an average length of 20.91 words.

The dataset encompasses eight imaging modalities, as summarized in Table 2. Computed Tomography (CT) (40,913) and Radiography/X-ray (31,827) constitute the majority, followed by Magnetic Resonance Imaging (MRI) (18,570) and Ultrasound (17,147). The smaller categories, namely Angiography (6055), Positron Emission Tomography (PET) (1134), combined PET/CT (580), and Optical Coherence Tomography (OCT) (409), were grouped under “Other” due to their limited distribution. This grouping was also adopted when developing a ResNet-50 [25] classifier used to predict image modality labels for lower-performing SVLMs at inference time.

Medical concept annotations include 1949 unique UMLS concepts in the training set, with smaller vocabularies in validation (716) and test (702) sets to support generalization assessment. The concepts span pathological findings (e.g., fluid accumulation, neoplasms, cysts), anatomical structures (e.g., cardiac, hepatic, cerebral regions), and imaging modalities categorized as diagnostic procedures (e.g., CT, X-ray, MRI), thus covering a broad clinical range for caption generation. The medical concepts constitute core components within captions that models learn to identify and integrate when generating structured outputs.

**Figure 1:**
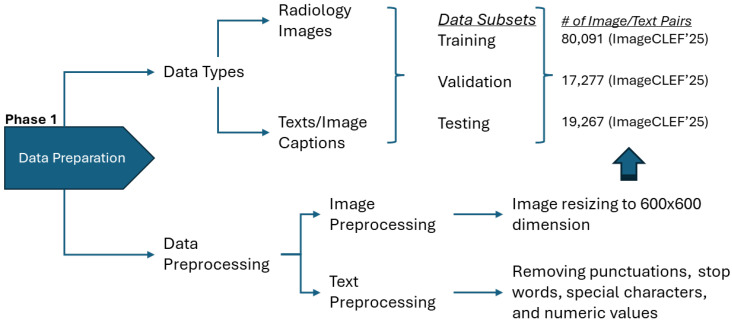
Data preparation pipeline showing image standardization and text normalization procedures.

Figure 1 illustrates the complete data preparation pipeline, encompassing image preprocessing to standardized 600 × 600 dimensions and text normalization procedures including punctuation removal and numeric token replacement. Collectively, this composition makes the dataset suitable for evaluating VLMs across multiple dimensions. Its modality diversity enables testing generalization across different imaging physics, caption variability challenges models to handle linguistic complexity, and the dataset’s size and structured partitioning provide a robust foundation for architectural comparisons.

### 2.2. Model Preparation

**Figure 2:**
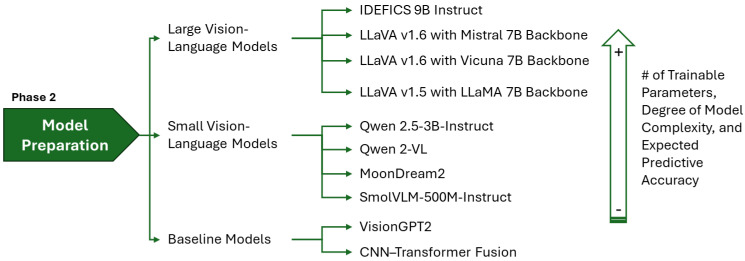
Model preparation taxonomy: categorization of LVLMs, SVLMs, and baseline architectures evaluated.

**Figure 3:**
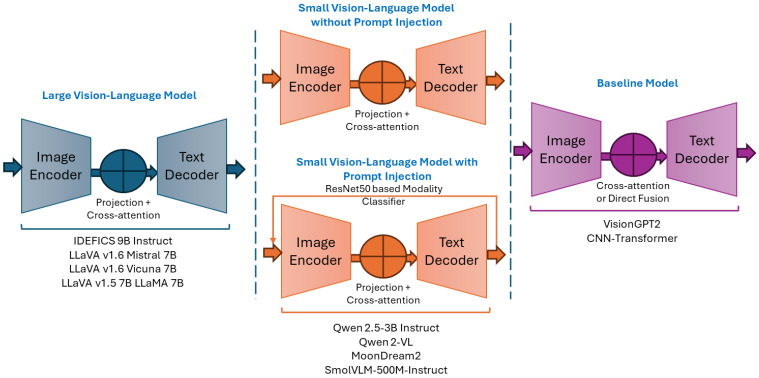
Conceptual frameworks of the corresponding LVLMs, SVLMs, and baseline architectures.

Multimodal learning for medical image captioning utilizes large model architectures that integrate medical imagery and clinical text generation [14,26,27]. The architectural landscape can be grouped into large models that support complex medical reasoning and compact models designed for efficiency. Our experimental framework included three categories of multimodal architectures, as shown in Figure 2 and summarized in Table 3. Figure 3 illustrates the conceptual frameworks of these architectures. The first group includes LVLMs with 7 B to 9 B parameters. The second group includes SVLMs with 0.5 B to 3 B parameters. The third group covers two baseline encoder–decoder architectures with fewer than 250 M parameters. Despite the differences in scale, VLMs [28] share core components for image captioning. Each uses a vision encoder for feature extraction, a language decoder for text generation, and a connector or fusion module for cross-modal alignment.

#### 2.2.1. Large Vision-Language Models (LVLMs)

All the variants of LLaVA architecture family use a CLIP ViT-L/14 vision encoder with approximately 430 M parameters, which processes input images through 24 transformer layers using 14×14 patch decomposition [29]. The vision encoder generates 576 visual tokens that capture spatial features at multiple scales. These features are then aligned with language representations through projection layers combined with cross-attention mechanisms (20 M to 50 M parameters, Table 3) to bridge the vision-language gap.

- **LLaVA v1.6 Mistral-7B** [29] pairs the CLIP ViT-L/14 encoder with Mistral-7B-Instruct as its language decoder. The Mistral backbone introduces Sliding Window Attention (SWA), which reduces computational complexity from $O(n^{2})$ to O(n×window) and supports a context length of 32,768 tokens. It also applies Grouped-Query Attention (32 query heads mapped to 8 key-value heads), lowering inference memory requirements by about 75% while preserving generation quality. A two-layer MLP connector (20 M to 50 M parameters) projects 1024-dimensional vision features into the 4096-dimensional language space, enabling cross-modal integration.
- **LLaVA v1.6 Vicuna-7B** substitutes Vicuna-7B as the language backbone while maintaining identical vision encoding and projection mechanisms. Vicuna-7B features 32 transformer layers with RMSNorm normalization and SwiGLU activation functions, supporting context lengths up to 4096 tokens [29]. Both Mistral and Vicuna variants use a similar training protocol. The models first align visual features with language embeddings using filtered image-caption pairs, and subsequently undergo visual instruction tuning.
- **LLaVA v1.5 with LLaMA-7B** features a LLaMA-7B [30] decoder containing 32 transformer layers utilizing RMSNorm normalization, SwiGLU activation functions, and Rotary Position Embeddings (RoPE). The architecture supports a 2048-token context window and incorporates standard multi-head self-attention with 32 heads, each operating on 128-dimensional representations. Cross-modal alignment is achieved through similar linear projection strategy, demonstrating that, with appropriate instruction tuning, complex fusion mechanisms are not required for effective performance.
- **IDEFICS-9B** [12] Instruct combines a CLIP ViT-H/14 vision encoder with LLaMA-7B. Its distinguishing feature is the Perceiver Resampler module containing approximately 250 M trainable parameters, which compresses variable-length visual inputs into exactly 64 visual tokens regardless of input size. The Perceiver operates through learned latent queries that extract fixed-size representations via cross-attention, reducing computational complexity to O(N×M) where N=64 latent tokens and *M* visual features. This design ensures a constant processing cost independent of image dimensions. Additionally, the model integrates Gated Cross-Attention Dense layers (approximately 500 M parameters) inserted every fourth transformer block, using tanh(α) gating to stabilize multimodal fusion during training.

#### 2.2.2. Small Vision-Language Models (SVLMs)

The emergence of SVLMs addresses the need for deployable multimodal systems in resource-constrained clinical environments. Recent advances in model compression and architectural efficiency have enabled these compact models to achieve competitive performance while requiring fewer computational resources than their larger counterparts [16]. These models typically range from 0.5 B to 3 B parameters, making them suitable for deployment in settings where computational infrastructure is limited [21].

- **Qwen 2-VL** [31] employs Native Dynamic Resolution (NaViT) processing, eliminating fixed-resolution constraints. The custom Qwen Vision Transformer (ViT) processes images at native resolutions, producing 4 to 16,384 visual tokens per image with 2D-RoPE positional encoding. A lightweight token merger aggregates spatially adjacent patches through MLP compression before language model fusion.
- **Qwen 2.5-3B-Instruct** [32] extends this architecture with window attention for computational efficiency and SwiGLU activations in vision MLPs, aligning encoder structure with modern language models. The PatchMerger component provides additional token compression through dedicated MLP sublayers. Notably, it implements Multimodal Rotary Position Embedding (M-RoPE), decomposing positions across temporal, height, and width dimensions for unified spatial–temporal reasoning. Both models support 32,768 token context windows, with Qwen 2.5-3B-Instruct offering enhanced positional encoding strategies optimized for variable-resolution multimodal processing.
- **MoonDream2** [29] combines SigLIP-base vision encoding with Phi-1.5 language model [33]. The architecture replaces CLIP’s softmax loss with pairwise sigmoid loss, eliminating global batch dependencies and improving zero-shot performance. The lightweight projection layer (per Table 3) requires minimal computational overhead while maintaining effective cross-modal alignment.
- **SmolVLM-500M-Instruct** [34] is the most compact model evaluated, employing aggressive visual token compression by reducing the number of visual tokens through pixel shuffle strategies. The Idefics3Connector projects high-dimensional vision features (12,288-dimensional) into a 960-dimensional language space, achieving approximately a 9× reduction in token representation while maintaining captioning quality.

#### 2.2.3. Baseline Architectures

- **VisionGPT2** combines a custom ViT [35] with GPT-2 [36] small as the text decoder. The ViT divides images into patches, applies positional embeddings, and extracts high-dimensional features. These features are fused into the GPT-2 decoder using multi-head cross-attention module.
- **CNN-Transformer Fusion** uses a pre-trained CheXNet [22] (DenseNet121) as the CNN feature extractor. A transformer encoder further contextualizes the extracted embeddings, which are then refined by a tiny transformer decoder [14]. The decoder generates captions through multi-head self-attention and cross-attention with the visual features. The architecture capitalizes on the spatial sensitivity of CNNs and the global sequence modeling power of transformers, linked by a parameter-efficient feature fusion layer.

The parameter distributions in Table 3 reveal underlying design philosophies, where LVLMs dedicate 75 % to 90% of parameters to language modeling, while SVLMs maintain more balanced vision-language distributions, directly impacting computational requirements from sub-1 GB (SmolVLM) to 32 GB+ (LVLMs) for deployment [37].

### 2.3. Training Environment Preparation

Traditional full fine-tuning of VLMs updates all parameters during training, which can be computationally prohibitive for models with billions of parameters. For instance, fully fine-tuning a 7 B parameter model requires substantial GPU memory (>32 GB) and risks catastrophic forgetting of pre-trained representations [38]. This is particularly problematic in medical domains, where preserving general visual-linguistic understanding alongside domain-specific knowledge is crucial [39]. PEFT [40] methods address these limitations by modifying only a small subset of model parameters while keeping the majority frozen. Recent studies demonstrate that PEFT achieves 95% to 98% of full fine-tuning performance while reducing trainable parameters by 99% and memory usage by up to 12-fold, making deployment viable in resource-constrained clinical settings [40]. Among PEFT techniques, LoRA has emerged as particularly effective for vision-language tasks [16].

LoRA decomposes weight updates into low-rank matrices, with adapted weights computed as follows:

(1) $$W=W_{0}+\Delta W=W_{0}+BA$$

where $W_{0}\in R^{d\times k}$ represents frozen pre-trained weights, $B\in R^{d\times r}$ (down-projection) and $A\in R^{r\times k}$ (up-projection) are trainable matrices with rank r≪min(d,k). The forward pass becomes as follows:

(2) $$h=W_{0}x+\frac{\alpha }{r}\;BAx,$$

Here, the rank *r* governs the adaptation’s capacity and parameter count, while the scaling factor α controls the update magnitude to stabilize training, with adapter scaling α/r modulating how strongly the LoRA layer adapts to new data [19].

### 2.4. Hardware and Software Environment

All experiments were conducted on Google Cloud Platform infrastructure with differentiated hardware allocation by model category. LVLMs (LLaVA variants, IDEFICS-9B) were trained on a2-highgpu-2g machines equipped with dual NVIDIA A100-40GB GPUs (24 vCPUs, 170 GB RAM), while SVLMs and baseline architectures utilized n1-highmem-16 machines with dual NVIDIA V100 GPUs (16 vCPUs, 104 GB RAM). The software environment comprised Python 3.12, PyTorch 2.2 with CUDA 12.1 for VLMs, and TensorFlow 2.16 for the CNN-Transformer baseline. Complete hardware and software specifications are provided in Appendix B, Table A1.

#### 2.4.1. Adaptation Strategy Selection

We evaluated three adaptation strategies to identify optimal configurations for medical image captioning:

1. **Targeted LoRA** focuses on core transformation layers, targeting the query (q), key (k), and value (v) projections within the attention mechanisms, the multimodal connector layers (mm_projector), and the gate (g), up (u), and down (d) projections of the MLP, while leaving the output projections and embedding layers unchanged. This configuration modifies only a tiny selected subset of overall model parameters.
2. **Extended LoRA** expands the adaptation to include output projections (o_proj) and the fully connected layers of MLP, increasing trainable parameters to a greater count.
3. **Hybrid Strategy** combines LoRA with full fine-tuning of language model head and token embeddings, reaching significantly greater trainable parameters and training time.

Preliminary experiments on representative architectures demonstrated that targeted LoRA consistently outperformed broader strategies, achieving superior Image-Caption Similarity (0.870 vs. 0.786 for extended) and better preserving medical terminology (UMLS F1: 0.154 vs. 0.129) of LLaVA-Mistral, as demonstrated in Section 3.1. Based on empirical optimization, we implemented differentiated configurations for the model categories defined in Table 4.

#### 2.4.2. Adaptive Full Fine-Tuning for Baseline Models

The baseline architectures employed progressive full fine-tuning to prevent catastrophic forgetting while adapting from general to medical-specific representations. For VisionGPT2, training began with the ViT encoder and GPT-2 decoder in a frozen state, updating only cross-attention modules that align image features with textual outputs. After initial progress, the encoder was unfrozen for joint tuning of image representations. The final phase activated the GPT-2 decoder for full-network refinement. The learning rate followed a OneCycleLR schedule [41], base value $1\times 10^{-4}$, with training in fp16 via GradScaler [42]. The effective batch size reached 32 by gradient accumulation. Data was augmented using random flipping, brightness, contrast, and rotation changes, while the CNN-Transformer Fusion sequence started with only the transformer decoder trainable and CheXNet (DenseNet121) fixed [22]. Once captions were stabilized, CNN layers became trainable for radiology-specific adaptation. The last stage fine-tuned all components together: CNN backbone, transformer encoder, and decoder. Scheduling ramped the learning rate to $1\times 10^{-4}$ in early steps. Sparse categorical cross-entropy loss incorporated padding mask and Vocabulary included 33,227 specialist and domain tokens.

Both baseline models monitored validation perplexity for early stopping (patience of 5 epochs) and saved best-performing checkpoints based on validation loss. This progressive approach proved essential for smaller architectures, enabling them to achieve reasonable performance despite full parameter updates.

#### 2.4.3. Training Configuration of Vision Language Models

**Figure 4:**
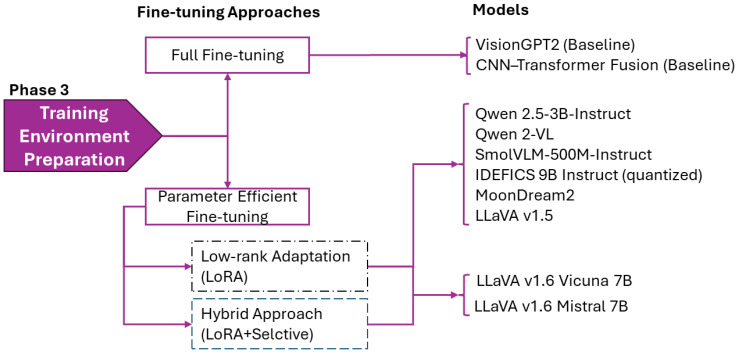
Training strategy allocation for baselines models and VLMs.

Training configurations were tailored to model capacity and memory constraints. All VLMs were fine-tuned with mixed precision training (bfloat16) [43] and Flash Attention 2 [44] to accelerate computation and reduce memory overhead during attention operations. AdamW Optimization [45] ($\beta_{1}$ = 0.9, $\beta_{2}$ = 0.999, ϵ = $1\times 10^{-8}$, weight decay = 0.01) was used with learning rates assigned as follows: $1\times 10^{-4}$ for LVLMs (LLaVA-v1.6-Mistral-7B, LLaVA-v1.6-Vicuna-7B, IDEFICS-9B-Instruct); $1\times 10^{-5}$ for LLaVA-1.5-7B; $5\times 10^{-5}$ for SmolVLM-500M; $3\times 10^{-4}$ for Qwen-2B and Qwen-2.5-3B; $3\times 10^{-6}$ for MoonDream2 (cosine scaling). Model progression was tracked using early stopping by observing validation loss. Patience was set to 3 evaluations for LVLMs and 6 for SVLMs. Typical convergence occurred within 3000 steps to 10,000 steps for the models. The training environment configuration, shown in Figure 4, enabled systematic evaluation across model families and comparison between parameter-efficient and full fine-tuning approaches.

#### 2.4.4. Modality-Aware Prompting for Small Vision Language Models

Given the performance gap between large and small VLMs observed in preliminary experiments, we investigated whether providing explicit modality information could enhance caption generation for under-performing SVLMs without requiring additional training. This approach reflects clinical practice where radiologists know the imaging modality before interpretation.

**ResNet-50 [25] based classifier** was employed for automated modality classification across five imaging modalities of ROCOv2 such as CT, MRI, Radiograph, Ultrasound, and Others. The *Others* category consolidated less frequent modalities including angiography, PET, PET/CT, and OCT, which collectively represented 7.0% of the dataset (Table 2). This grouping addressed the class imbalance that would have resulted from treating these infrequent modalities independently. The architecture utilized standard ResNet-50 topology without ImageNet pretraining, ensuring features were learned exclusively from medical images [46]. The final fully connected layer was modified to produce five outputs corresponding to the target modalities. Each modality class was downsampled to 8178 images to match the smallest consolidated class size, addressing dataset class imbalance.The classifier underwent training using cross-entropy loss [47] and Adam optimization [48]. Its validation metrics demonstrated robust performance with accuracy of 96.46%, and balanced precision, recall, and F1-scores all approximating 0.964. Distributional analysis of test set predictions confirmed balanced outputs across all five modalities.

**Inference-Time Implementation:** The modality-aware prompting strategy was implemented as a two-stage inference pipeline exclusively for the three underperforming SVLMs (SmolVLM-500M, Qwen 2-VL, and Qwen-2.5-3B). In the first stage, the trained ResNet-50 [25] classifier predicted the imaging modality for each test image. In the second stage, during caption generation, the predicted modality label was prepended to the input prompt using the template: “[Modality] image: Describe the medical image.” This resulted in prompts such as “CT image: Describe the medical image” or “MRI image: Describe the medical image.” The modality-conditioned prompting aimed to guide models toward appropriate domain-specific vocabulary and structural patterns characteristic of each imaging type.

**Design Considerations:** Modality information was incorporated solely during inference, not during model training. This design preserved modularity, as captioning models retained their functionality without architectural modifications or retraining. The decision to exclude modality prompting from LVLMs was based on their stronger baseline performance metrics, suggesting adequate implicit modality understanding. The inference-only augmentation strategy provided a practical approach to potentially enhance performance while maintaining system simplicity and the option to disable prompting if adverse effects were observed.

### 2.5. Post-Training Evaluation

**Figure 5:**
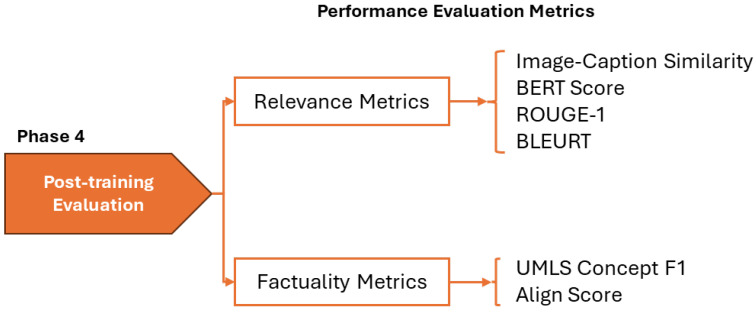
Evaluation framework with relevance metrics used to assess caption quality.

The quality of generated captions for the test images was evaluated using a comprehensive suite of metrics to measure relevance to the reference caption and clinical factuality. This evaluation framework, introduced in ImageCLEFmedical 2025 [49] and illustrated in Figure 5, computes individual metric scores for each caption that are then averaged over the test set. Three aggregate measures are reported: Relevance Average (mean of four relevance metrics), Factuality Average (mean of two factuality metrics), and Overall Average (mean of all six metrics), providing a single summary score across relevance and factuality. By incorporating measures that capture lexical overlap, semantic alignment, image–text correspondence, and concept-level accuracy, the evaluation provides a balanced assessment of caption quality and clinical utility.

#### 2.5.1. Relevance Metrics

Relevance is quantified using four complementary approaches, which are described below.

1. **Image-Caption Similarity**: It is computed by mapping the image and candidate caption into a shared embedding space using MedImageInsight [50], a medical VLM trained across X-ray, CT, MRI, OCT, and ultrasound modalities, and calculating their cosine similarity. This metric assesses whether the caption content aligns with the visual signal without requiring reference text.
2. **BERTScore**: It is applied in its recall-oriented configuration with inverse document frequency weighting, following established best practices for caption evaluation [51]. Contextual embeddings were generated using a DeBERTa-XLarge-MNLI encoder [52], selected for its demonstrated correlation with human quality judgments.
3. **ROUGE-1**: F-measure calculated to assess lexical overlap through unigram matching between the candidate and reference captions [53].
4. **BLEURT**: it is applied using the BLEURT-20 checkpoint to obtain learned quality scores that approximate human preferences for caption quality [54].

To minimize formatting artifacts and focus evaluation on semantic content, captions for BERTScore, ROUGE, and BLEURT underwent preprocessing including lowercasing, punctuation removal, numeric token replacement with “number,” and single-sentence treatment regardless of original segmentation.

#### 2.5.2. Factuality Metrics

Clinical factuality was assessed using two metrics targeting correctness at different levels of granularity.

1. **UMLS Concept F1**: It quantified preservation of key medical entities through comparison of concept sets extracted from candidate and reference captions. Medical concepts were identified using MedCAT [55] and filtered according to clinically relevant semantic types specified in the MEDCON framework [56].
2. **AlignScore**: It generated consistency scores by evaluating factual claims in candidate captions against reference standards using a RoBERTa-based alignment model [57]. The metric decomposes captions into claims and aligns them with supporting evidence, producing averaged alignment scores.

The evaluation metrics balance surface overlap, semantic similarity, image–text alignment, and concept-level correctness, which together are important for caption usefulness in radiology. The final ranking of systems relied on the Overall Average, with Relevance Average and Factuality Average reported to separate linguistic similarity from clinical accuracy. Framing the evaluation in this way facilitates comparison not only across different model families but also between fine-tuning strategies. It establishes a standardized protocol for assessing caption quality, making the reported results interpretable beyond the scope of a single dataset or task.

## 3. Experimental Results

### 3.1. Optimal Adaptation Strategy Selection

Before conducting cross-model comparisons, we evaluated LoRA strategies to identify optimal configurations for parameter-efficient fine-tuning. Using the LLaVA architecture with two different language model backbones (Mistral-7B and Vicuna-7B) as representative examples, we compared adaptation approaches varying in complexity and parameter count. For LLaVA Mistral-7B, the following were our evaluations: (1) LoRA selectively applied to attention mechanisms and MLP blocks (40.1 M parameters, 0.53% of model), (2) extended LoRA including output projections and multimodal connector layers (98.4 M parameters, 1.28%), and (3) a hybrid approach combining LoRA with full fine-tuning of language head and embeddings (350.6 M parameters, 4.58%). For LLaVA Vicuna-7B, we compared targeted LoRA (34.4 M parameters, 0.48%), including multimodal projections, against the hybrid approach (346.7 M parameters, 4.85%). The layer targets for each configuration are provided in the methodology.

**Figure 6:**
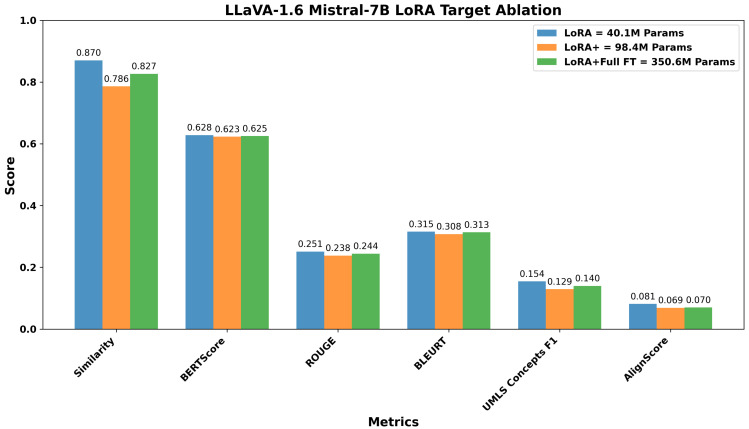
Comparison of targeted, extended, and hybrid adaptation strategies for LLaVA-1.6 Mistral-7B across six evaluation metrics. The targeted approach, with 40.1 M trainable parameters (0.53%), achieves the highest image-caption similarity and shows competitive performance on BERTScore, ROUGE, BLEURT, and the factuality metrics (UMLS Concepts F1 and AlignScore).

**Figure 7:**
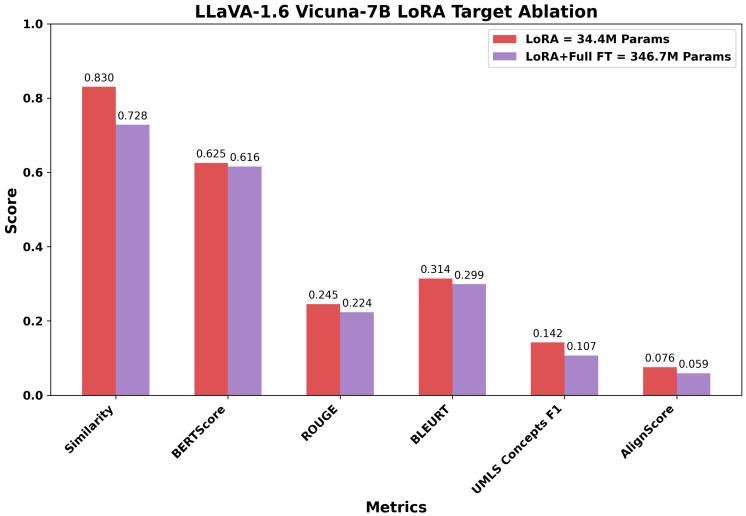
Comparison of targeted and hybrid adaptation strategies for LLaVA-1.6 Vicuna-7B across six evaluation metrics. The targeted LoRA, with 34.4 M trainable parameters (0.48%), consistently achieves stronger image-caption similarity and higher scores in BERTScore, ROUGE, BLEURT, as well as improved factuality (UMLS Concepts F1 and AlignScore), compared to the hybrid strategy that trains ten times more parameters.

Figure 6 and Figure 7 show the results across six evaluation metrics. For LLaVA Mistral-7B, the targeted LoRA achieved the highest Image Caption Similarity score of 0.870, compared to 0.786 for extended LoRA and 0.827 for the hybrid approach, corresponding to relative drops of 9.7% and 5.0%, respectively. A similar trend was observed across relevance-oriented metrics, with BERTScore showing minimal variation (0.628, 0.623, 0.625) but ROUGE and BLEURT exhibiting clearer advantages for targeted adaptation. ROUGE scores decreased from 0.251 (targeted) to 0.238 (extended) and 0.244 (hybrid), while BLEURT scores showed a similar pattern, declining from 0.315 (targeted) to 0.308 (extended) and 0.313 (hybrid).

The performance differences were more pronounced in factuality metrics. UMLS Concepts F1, which measures the preservation of medical terminology, decreased from 0.154 (targeted) to 0.129 (extended) and 0.140 (hybrid), representing 16.2% and 9.1% reductions. Similarly, AlignScore dropped from 0.081 to 0.069 (14.8% reduction) and 0.070 (13.6% reduction), respectively. These metrics are clinically salient because they reflect preservation of medical terminology and factual consistency.

The LLaVA Vicuna-7B results reinforced this pattern. The LoRA configuration (34.4 M parameters) outperformed the hybrid approach (346.7 M parameters) despite using one-tenth of the trainable parameters. Image Caption Similarity decreased from 0.830 to 0.728 (12.3% reduction), while UMLS Concepts F1 dropped from 0.142 to 0.107 (24.6% reduction) and AlignScore fell from 0.076 to 0.059 (22.4% reduction). The substantial degradation in factuality metrics with expanded adaptation complexity further suggests that modifying larger parameter sets can disrupt the model’s learned medical knowledge representations rather than enhancing them.

Overall, increasing the number of trainable parameters does not necessarily improve performance across relevance and factuality dimensions. The targeted LoRA approach, which modifies only a small and selective subset of the pre-trained model’s parameters, achieved comparable or superior task-specific adaptation. This suggests that modest and targeted intervention in the model’s parameter space is sufficient to retain general pre-trained representations while enabling effective incorporation of domain-specific knowledge. Consequently, all subsequent cross-model comparisons report results for the targeted adaptation variant of each VLM, favoring configurations that balance performance with computational efficiency.

### 3.2. Cross-Model Performance Comparison

Having established that targeted LoRA provides optimal performance-efficiency trade-offs, we evaluated eight vision-language models and two baseline architectures on the radiology image captioning task. All models employed their best configurations as determined by preliminary experiments. Table 5 presents comprehensive performance metrics across all evaluated systems.

The results reveal a performance hierarchy across model categories. Among LVLMs, LLaVA Mistral-7B achieved the highest overall average (0.317), with particularly strong performance in Image-Caption Similarity (0.870) and Relevance Average (0.516). The model substantially outperformed the strongest baseline architecture (VisionGPT2: 0.177 overall average) while using only 0.53% trainable parameters compared to the baseline’s full fine-tuning approach. The systematic ablation study demonstrated that this performance advantage remained consistent across adaptation strategies. LLaVA Vicuna-7B followed closely with an overall average of 0.306, showing comparable performance with even fewer trainable parameters (0.48%). IDEFICS-9B achieved intermediate performance (0.290 overall), while LLaVA-1.5 showed lower scores (0.273), likely reflecting its older architecture and training setup.

Among SVLMs, MoonDream2 emerged as the strongest performer with an overall average of 0.279, attaining a performance comparable to that of LLaVA-1.5 (0.273) while using approximately 74% fewer total parameters. Notably, MoonDream2’s relevance average (0.466) approached that of some LVLMs, with its Image Caption Similarity (0.757) and BLEURT scores (0.303) demonstrating competitive semantic alignment. Moreover, MoonDream2’s factuality metrics (0.093) also outperformed LLaVA-1.5 (0.083), suggesting that architectural innovations and training strategies can partially compensate for reduced model size. However, the top-performing LLaVA Mistral-7B maintains clear advantages with factuality scores of 0.118, indicating that while efficient architectures narrow the gap, fundamental capacity differences remain for the most demanding clinical applications. Qwen 2-VL, SmolVLM, and Qwen-2.5 showed progressively lower performances, with 0.232, 0.200, and 0.188 average overall scores respectively. Interestingly, Qwen-2.5 achieved the lowest overall average (0.188) among SVLMs, even underperforming SmolVLM (0.200), which has fewer total parameters (0.5 B vs. 3.1 B). This counterintuitive result suggests that model architecture and pre-training quality may be more determinative than raw parameter count for smaller models. Qwen 2-VL presents another anomaly, achieving the highest AlignScore (0.109) among all models including LVLMs while having a relatively weaker overall performance (0.232). This unusually strong factual claim consistency despite weak relevance metrics (0.372) suggests that Qwen 2-VL may have been pre-trained on data distributions that emphasize factual alignment over semantic similarity, though this comes at the cost of overall caption quality.

The baseline models performed substantially below all LoRA-adapted VLMs. VisionGPT2, the stronger baseline with an overall average of 0.177, was outperformed by even the lowest performing SVLM (Qwen-2.5 at 0.188). The CNN-Transformer baseline showed the weakest performance (0.154 overall), with particularly poor ROUGE scores (0.044) indicating limited n-gram overlap with reference captions. These results demonstrate that parameter-efficient adaptation of pre-trained VLMs consistently outperforms traditional fine-tuned baselines, even when the LVLMs and SVLMs use less than 1% and 9% of the total trainable parameters, respectively. The evaluation represents single runs per model configuration; while the multi-dimensional consistency across multiple architectures and adaptation strategies provides evidence of systematic advantages, formal assessment of variance across random seeds would strengthen confidence in these findings.

**Figure 8:**
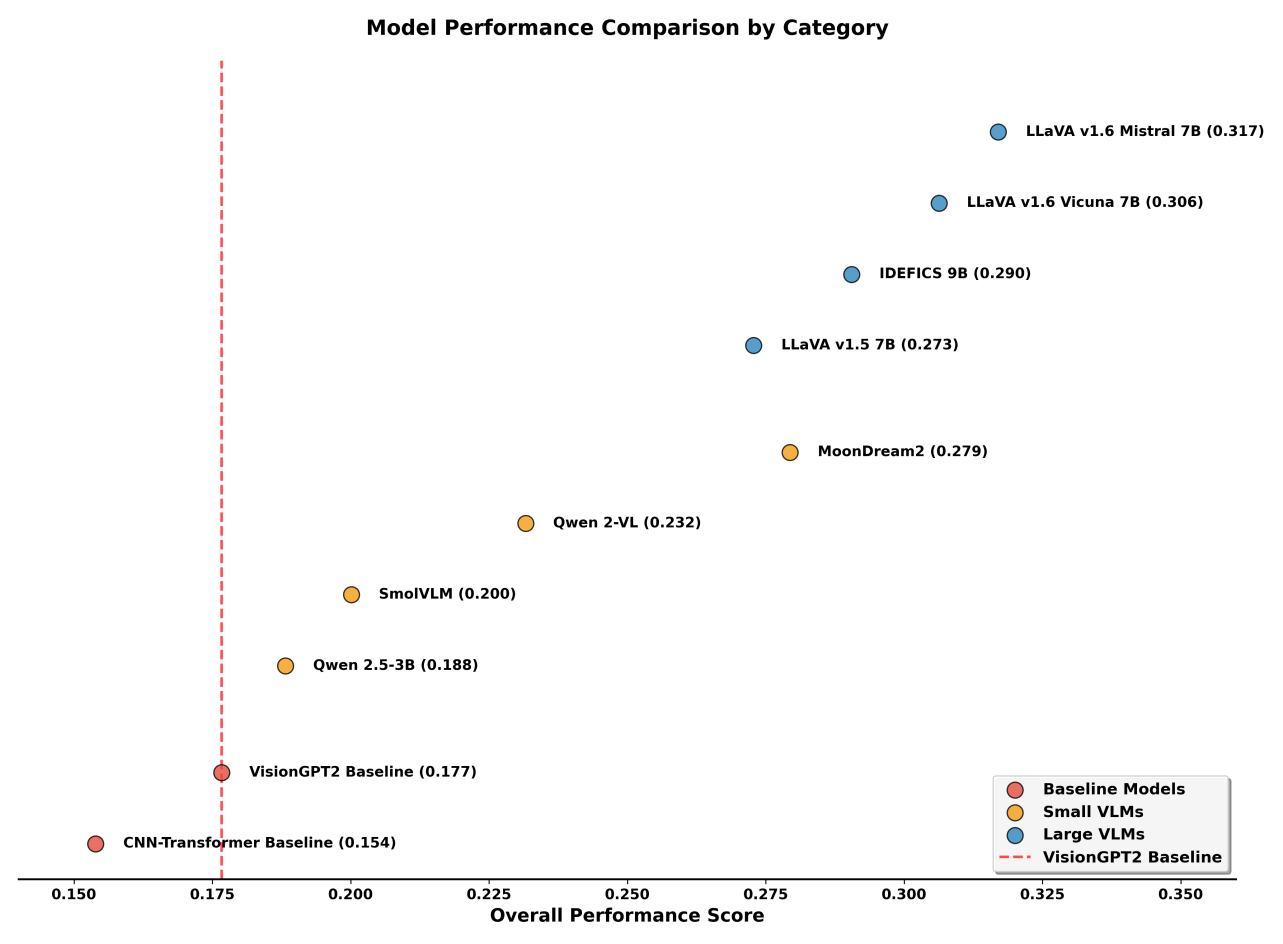
Overall performance scores across model categories. LVLMs (blue) and SVLMs (orange) consistently outperform baseline models (red). The dashed line indicates the VisionGPT2 baseline performance (0.177), showing all LoRA-adapted models exceed this threshold. While LVLMs generally achieve higher performance than SVLMs, MoonDream2 represents a notable exception, achieving a performance comparable to that of LLaVA-1.5 despite having approximately 74% fewer parameters.

Figure 8 illustrates the performance distribution across model categories by overall average. There is a distinctive separation between LVLMs (0.273 to 0.317 range), SVLMs (0.188 to 0.279 range), and baselines (0.154 to 0.177 range). Notably, MoonDream2 bridges the gap between small and large models, achieving a performance comparable to that of LLaVA-1.5 despite its significantly smaller size.

**Figure 9:**
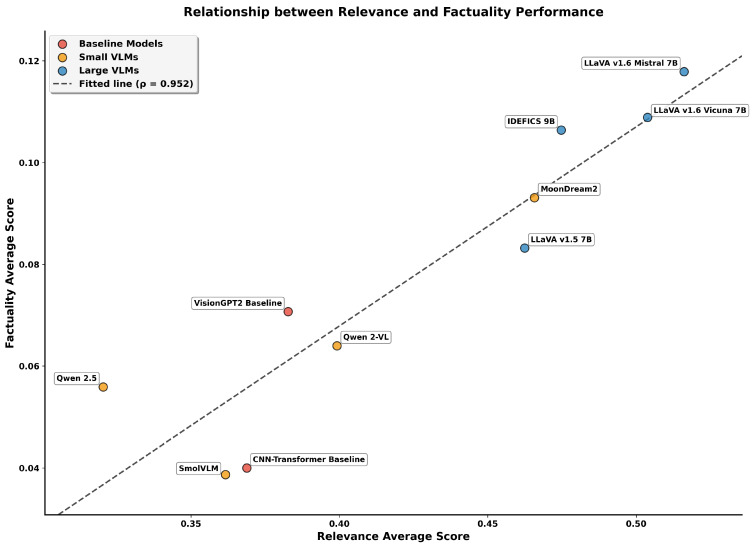
Relationship between relevance and factuality performance across models. LVLMs (blue) cluster in the upper-right quadrant demonstrating superior performance on both dimensions, while baseline models (red) occupy the lower-left region. SVLMs (orange) show intermediate and variable performance. The fitted line is shown for visual reference; analysis is provided in Appendix A.

To examine how linguistic relevance relates to clinical factuality, Figure 9 presents the relationship between Relevance Average and Factuality Average across all evaluated models. The scatter plot reveals distinct clustering patterns by model category, with additional analysis provided in Appendix A.

The scatter plot reveals distinct clustering patterns. LVLMs occupy the upper-right quadrant (relevance 0.462 to 0.516, factuality 0.083 to 0.118), while baselines cluster in the lower-left region (relevance 0.284 to 0.325, factuality 0.024 to 0.028). SVLMs show greater variability between these extremes. MoonDream2 represents a particularly interesting case, achieving relevance comparable to that of IDEFICS-9B (0.466 vs. 0.482) while maintaining factuality (0.093) that exceeds that of LLaVA-1.5 (0.083). This performance profile, where a 1.86 B parameter MoonDream2 matches or exceeds certain 7 B+ models on both dimensions, demonstrates that model efficiency can be achieved without proportional sacrifices in caption quality. These findings establish distinct performance tiers for clinical deployment consideration. The top-performing LVLMs (LLaVA Mistral-7B and Vicuna-7B) are suitable for diagnostic applications requiring high accuracy. MoonDream2 occupies a unique position, offering a performance that rivals some LVLMs while maintaining computational efficiency that could support deployment in resource-constrained environments, although integration into screening workflows would require validation of timing, accuracy, and human–AI interaction patterns in clinical settings. The remaining SVLMs show mixed capabilities, with Qwen 2-VL’s strong factual alignment but weak relevance, suggesting specialized use cases where claim consistency is prioritized over semantic fluency. Baseline models, with overall averages below 0.18, demonstrate the clear advantages of modern VLM architectures over traditional approaches.

### 3.3. Modality-Aware Prompting for Performance Enhancement in SVLMs

Given the performance gap between SVLMs and LVLMs, we investigated whether providing explicit modality information at inference time could enhance SVLM performance. In clinical practice, radiologists always know the imaging modality before interpretation, suggesting this contextual information could help models generate more appropriate captions. To test this hypothesis, we trained a ResNet-50 classifier on our dataset to predict image modality (CT, MRI, Radiograph, Ultrasound, Other), and then prepended these predicted labels to the input during inference for three low-performing SVLMs: SmolVLM, Qwen 2-VL, and Qwen-2.5-Instruct.

Table 6 shows that modality-aware prompting produced mixed results. SmolVLM improved slightly, with the overall score rising from 0.200 to 0.207, which is an increase of 3.5%. The largest changes were in factuality metrics: UMLS F1 rose from 0.016 to 0.031, an increase of 93.8%, and AlignScore increased from 0.060 to 0.096, a gain of 60.0%. Although these percentages are large, the absolute scores remain very low, which limits their clinical significance. Qwen-2.5 also showed modest improvement, with the overall score increasing from 0.188 to 0.200, equivalent to a 6.4% gain. This improvement was primarily in relevance, where the average increased from 0.320 to 0.351 (9.7%), supported by gains in Image Caption Similarity from 0.449 to 0.502 (11.8%). However, factuality declined from 0.056 to 0.049, a reduction of 12.5%.

In contrast, Qwen 2-VL experienced relative declination in performance. Its overall score decreased from 0.232 to 0.182, a reduction of 21.6%. Both relevance and factuality dropped sharply: Image Caption Similarity fell from 0.570 to 0.364, representing a 36.1% decrease, while UMLS F1 fell from 0.074 to 0.017, a reduction of 77.0%.

These divergent outcomes across the three models are consistent with recent findings on prompt sensitivity in VLMs, where explicit contextual information can yield variable effects depending on model architecture and training [58]. Understanding these mechanisms through sensitivity analysis evaluating how SVLMs respond to correctly versus incorrectly predicted modality labels would provide valuable insights for optimizing inference-time interventions and enabling model-specific adaptation strategies.

Taken together, these results show that modality-aware prompting cannot bridge the gap between SVLMs and LVLMs. At best, SmolVLM and Qwen-2.5 gained modest improvements, but their absolute scores remain well below those of even the weakest LVLM. The performance decline of Qwen 2-VL further illustrates the risks of inference-time interventions without model-specific validation. Overall, providing image modality during caption prediction at inference time may offer small benefits in certain cases. However, it cannot substitute the architectural advantages of LVLMs and does not represent a reliable strategy for enhancing SVLMs in clinical applications.

## 4. Discussion

VLMs offer potential as automated interpretation tools to accelerate report generation through clinically meaningful image captions. In this study, we compared LVLMs and SVLMs fine-tuned on the ROCOv2 dataset to assess their clinical utility. The comprehensive evaluation of VLMs for radiology image captioning reveals findings that merit closer examination on model scaling and adaptation strategies. Performance varied by model scale, with larger architectures generally outperforming smaller ones. However, lightweight adaptation using LoRA proved effective across the models.

Additionally, our investigation of inference-time interventions revealed that modality-aware prompting cannot compensate for fundamental architectural limitations in small models. While marginal improvements were observed for SmolVLM and Qwen-2.5, the absolute performance gains remained clinically insignificant. Moreover, simple radiologic findings were accurately captured by most models, while complex cases requiring multiple abnormality detection revealed clear performance stratification. These observations inform deployment strategies, with different model categories suitable for distinct clinical tasks ranging from initial abnormality detection to comprehensive diagnostic reporting. Overall, the study findings guide the selection of VLMs for resource-constrained medical imaging applications.

### 4.1. The Parameter Efficiency Paradox

Contrary to intuitive expectations, targeted LoRA using 0.48% to 0.53% of model parameters outperformed hybrid approaches that modified up to 4.85% of parameters. The results become clearer when examining the specific components targeted by each approach. Targeted adaptation focused on attention mechanisms and MLP layers while preserving output projections and embedding layers intact. Hybrid approaches additionally performed full fine-tuning on language heads and embeddings, which appears to disrupt pre-trained knowledge representations.

The preservation of medical terminology, evidenced by higher UMLS Concept F1 scores in selective adaptation for LLaVA Mistral-7B and LLaVA Vicuna-7B, suggests that targeted parameter modification maintains semantic understanding essential for clinical applications. Recent work in medical NLP has similarly demonstrated that extensive parameter modification increases catastrophic forgetting, particularly when target domains diverge substantially from pre-training distributions [59]. In medical imaging, where both visual patterns and terminology differ markedly from natural images, preserving foundational knowledge while introducing focused adaptations appears superior to aggressive fine-tuning strategies.

### 4.2. Performance Patterns Across Caption Complexity

**Figure 10:**
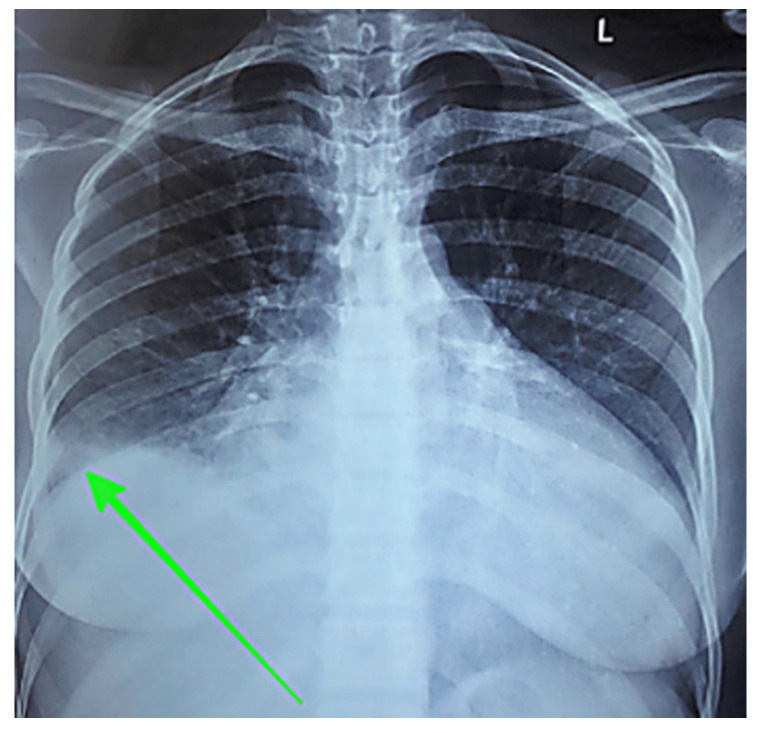
Representative chest X-ray example from the test set with green arrow indicating a right-sided pleural effusion.

Two representative test cases from the dataset’s most prevalent modalities (i.e., *CT scan* and *X-ray scan*) demonstrate how model capabilities translate to real-world performance. The chest X-ray case (Figure 10) illustrates an example of the nature of model performance. Table 7 presents detailed component analysis for this example. LVLMs generally identified core components such as image modality, anatomical location, and pathological findings with high fidelity for simple cases [11]. However, multiple LVLMs also introduced the descriptor *large*, which was absent from the ground truth. Additionally, most models correctly identified laterality (right-sided) and reproduced visual markers (parenthetical arrow descriptions), though these elements aid clinical interpretation but are not represented as UMLS concepts. This pattern appeared in other test cases as well, indicating a tendency of these models to amplify the perceived prominence of findings. Among SVLMs, performance varied considerably. MoonDream2 retained most components accurately despite the simplified visual marker. In contrast, Qwen 2.5 failed entirely by misidentifying both modality (*CT* instead of *X-ray*) and anatomy (*kidney* instead of *chest*), despite having a greater parameter count than Qwen 2-VL and SmolVLM.

**Figure 11:**
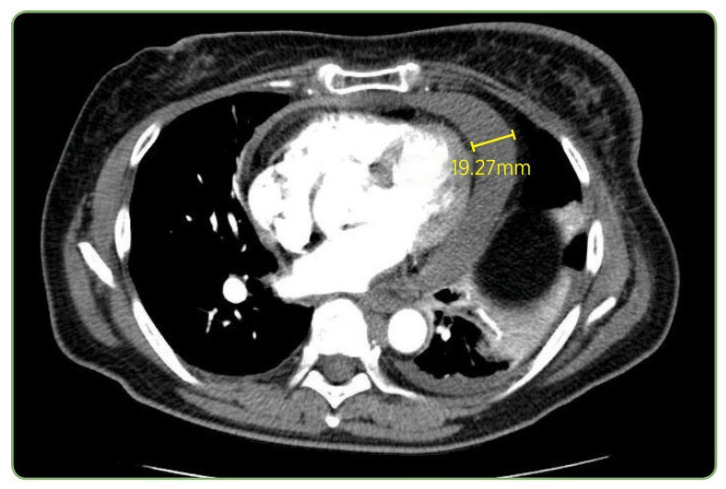
Representative CT pulmonary embolus study from the test set showing pericardial effusion measuring 19.27 mm.

The CT case in Figure 11 and Table 8 exposed more pronounced performance stratification. Only LLaVA-Mistral-7B reproduced all components including the exact measurement *(19.27 mm)*. LLaVA-Vicuna-7B also captured the pathological finding and measurement correctly, although it substituted *pulmonary angiogram* for *pulmonary embolus study*, which is a variation in clinical terminology. LLaVA-1.5 and IDEFICS-9B correctly identified both the modality and the pulmonary context, matching the study type, though neither detected the actual pathological finding of *pericardial effusion*. LLaVA-1.5 incorrectly described a *pulmonary embolism* in the right lower lobe as the primary finding, while IDEFICS-9B produced a truncated and incomplete description.

Among SVLMs, MoonDream2 correctly identified the modality and general anatomical region (*chest*) but misinterpreted the finding as a *large mass in the right upper lobe*. Qwen 2-VL correctly identified the modality but described a vague *right-sided mass* without proper anatomical context. Both failed to identify the *pericardial effusion*. Qwen 2.5 failed completely by mislabeling the study as an *axial T2 MRI*. SmolVLM demonstrated partial success by correctly identifying the modality, chest anatomy, and extracting the precise measurement *19.27 mm*, though it completely misattributed the measurement’s clinical context, suggesting surface-level pattern matching without semantic understanding. Thus, the performance degradation in Qwen 2-VL when modality prompting was applied underscores the need for careful validation before implementing augmentation strategies in clinical workflows. Analysis of caption generation across varying complexity levels exposed consistent patterns in model behavior.

The baseline models showed a mixed performance. VisionGPT2 partially identified the modality (*CT* component of *PET/CT*) but misidentified the anatomy as *hepatic*. CNN-Transformer correctly identified the modality but described an incorrect finding (*liver lesion*). These fundamental errors in pathological finding identification underscore the limitations of traditional architectures even with full fine-tuning.

Overall, the LVLMs outperform SVLMs, which, in turn, exceed the fully fine-tuned baseline models. This performance stratification was evident in both component-level accuracy and overall caption coherence, as demonstrated in the two representative cases. The pattern holds across both simple and complex cases, with LVLMs achieving Overall Average scores of 0.273 to 0.317, SVLMs scoring 0.188 to 0.279, and baselines at 0.154 to 0.177. However, within each model family, performance comparisons become more nuanced. Among LVLMs, LLaVA-Mistral-7B outperformed IDEFICS-9B despite being smaller, while in the SVLM category, MoonDream2 exceeded Qwen 2.5 by a substantial margin. These intra-family variations indicate that when comparing models of similar scale, architectural design choices and pre-training quality become more determinative of performance than parameter count alone.

### 4.3. MoonDream2: Bridging the Efficiency Gap

In addition, MoonDream2’s performance profile demonstrates that efficient architectures can bridge the gap between model size and capability. It achieved relevance scores (0.466) comparable to IDEFICS-9B (0.482) and exceeded LLaVA-1.5 (0.462) despite being approximately 74% smaller than LLaVA-1.5. This efficiency likely stems from architectural optimizations in its SigLIP vision encoder and Phi-1.5 language backbone. Although MoonDream2 failed on complex multi-component captions, its reasonable performance on simpler cases suggests potential applicability in screening contexts prioritizing basic abnormality detection over detailed characterization, subject to validation in clinical pilot studies with radiologist oversight.

## 5. Conclusions and Future Work

Parameter-efficient fine-tuning of VLMs demonstrates technical feasibility as a foundation for future development of automated radiology reporting systems in clinical settings. Our empirical evaluation on 116,635 radiological images demonstrates that targeted LoRA, modifying less than 1% of model parameters, outperforms both extended adaptation strategies and traditional fully fine-tuned architectures. LLaVA-Mistral-7B with LoRA achieved the highest performance metrics (Relevance: 0.516, Factuality: 0.118), establishing a benchmark for medical image captioning. The observed performance hierarchy across model scales provides empirical evidence to inform future implementation strategies. LVLMs demonstrate optimal performance on the ROCOv2 benchmark where caption accuracy is paramount. These outcomes highlight that model design and efficient tuning can offset scale limitations, establishing benchmarks for subsequent workflow integration studies.

The benchmarks established in this study provide empirical foundations for developing AI-assisted radiology systems that enhance diagnostic efficiency while maintaining clinical accuracy. However, the evaluation relies on the ROCOv2 dataset, which may not capture all clinical scenarios, particularly given the dataset’s distribution toward CT, X-ray, MRI, and ultrasound images. The absence of radiologist evaluation limits understanding of clinical utility, as automated metrics may not fully capture diagnostic relevance. The work focused solely on English captions, excluding potential multilingual applications valuable in global healthcare contexts. Future work should integrate radiologist evaluation, investigate more complex datasets with greater modality diversity, incorporate modality-stratified analysis with statistical variance measures, and explore ensemble approaches that combine architectural strengths. As healthcare systems face increasing imaging volumes with constrained radiologist resources, the performance and computational characteristics of parameter-efficient VLM adaptation suggest promise for future systems designed to augment clinical expertise and standardize diagnostic reporting. Translation to clinical practice will require prospective validation studies that address workflow integration, human–machine interaction protocols, and safety considerations specific to each deployment context.

## Figures and Tables

**Table 1:** Summary of current study’s extensions from our preliminary investigation [14].

Component	Previous Work [14]	Current Work
**Model Coverage**		
Architectures evaluated	2	10
LVLMs (7 B to 9 B)	1	4
SVLMs (0.5 B to 3.1 B)	0	4
Baseline models	1	2
**Adaptation Strategy Analysis**		
Ablation study	Not performed	Section 3.1
LLaVA-Mistral strategies compared	Single config	3 strategies
LLaVA-Vicuna strategies compared	Not evaluated	2 strategies
Parameter range explored	40.1 M only	22 M to 350.6 M
**Evaluation Framework**		
Individual metrics used	10 metrics	8 metrics
Metric organization	Unstructured	Systematic
Composite metric categories	None	3 new
Relevance Metric	Not defined	BERTScore, ROUGE, BLEURT, Similarity
Factuality Metric	Not Available	UMLS Concept F1, AlignScore
**Experiments and Analysis**		
Parameter efficiency study	Not included	Section 4.1
Modality-aware prompting	Not explored	Section 3.3 (ResNet-50)
Qualitative case studies	Not included	Section 4.2

**Table 2:** Distribution of imaging modalities in the ROCOv2 dataset.

Primary Modalities	Count	Percentage
CT	40,913	35.1%
X-ray	31,827	27.3%
MRI	18,570	15.9%
Ultrasound	17,147	14.7%
Other *	8178	7.0%

* Other includes Angiography, PET, PET/CT, and OCT.

**Table 3:** Comparison of VLMs: encoders, decoders, connectors, and parameters.

Model	Image Encoder	Text Decoder	Connector/Fusion	# Params (Approx.)
IDEFICS 9B Instruct	CLIP ViT-H/14 (1.3 B)	LLaMA-7B (7.0 B)	Perceiver + gated cross-attn (0.75 B)	9.0 B
LLaVA v1.6 Mistral 7B	CLIP ViT-L/14 (∼0.43 B)	Mistral-7B (∼7.0 B)	Projection + cross-attn (∼20 M–50 M)	7.6 B
LLaVA v1.6 Vicuna 7B	CLIP ViT-L/14 (∼0.43 B)	Vicuna-7B (∼6.7 B)	Projection + cross-attn (∼20 M–50 M)	7.1 B
LLaVA v1.5 with LLaMA 7B	CLIP ViT-L/14 (∼0.43 B)	LLaMA-7B (∼6.7 B)	Projection + cross-attn (∼20 M–50 M)	7.1 B
Qwen 2.5-3B-Instruct	Custom ViT (∼0.3 B)	Qwen LM (∼2.8 B)	Projection + cross-attn (∼50 M)	3.1 B
Qwen 2-VL	Custom ViT (∼0.3 B)	Qwen LM (∼1.9 B)	Projection + cross-attn (∼20 M)	2.2 B
MoonDream2	SigLIP-base (∼0.15 B)	Phi-1.5 (∼1.7 B)	Projection + cross-attn (∼10 M)	1.86 B
SmolVLM-500M-Instruct	SigLIP-base (∼0.15 B)	Tiny LM (∼0.35 B)	Projection + cross-attn (∼10 M)	0.5 B
VisionGPT2	ViT (∼0.05 B)	GPT2 small (∼0.12 B–0.15 B)	Cross-attention (∼5 M–10 M)	0.21 B
CNN–Transformer Fusion	Tiny CNN (∼10 M)	Tiny Transformer (∼30 M–35 M)	Direct feature fusion (∼3 M)	0.048 B

**Table 4:** LoRA configuration parameters of the models.

Model	Params Trained	% of Total	Adapter Scaling	Target Modules
**LVLMs**				
LLaVA-Mistral-7B	40.1 M	0.53	2.0	q,k,v + MLP
LLaVA-Vicuna-7B	34.4 M	0.48	1.0	q,k,v + MLP + mm_proj
LLaVA-1.5	84.6 M	1.18	1.0	q,k,v,o + MLP + mm_proj
IDEFICS-9B	22.0 M	0.24	2.0	q,k,v only
**SVLMs**				
Qwen-2.5-3B	57.0 M	1.87	0.5	All attention + MLP
Qwen-2-VL	54.0 M	2.46	1.0	All attention + MLP
MoonDream2	74.4 M	3.85	0.25	All linear + proj
SmolVLM-500M	41.7 M	8.34	2.0	All linear
**Baselines**				
VisionGPT2	210 M	100	–	Progressive full FT
CNN-Transformer	48 M	100	–	Progressive full FT

**Table 5:** Performance comparison of vision-language models and baseline architectures.

Model	Parameters Trained (%)	Similarity	BERTScore	ROUGE	BLEURT	UMLS Concept F1	AlignScore	Relevance	Factuality	Overall
**Large VLMs (LoRA Adapter)**										
LLaVA Mistral-7B	0.53	0.870	0.628	0.251	0.315	0.154	0.081	0.516	0.118	0.317
LLaVA Vicuna-7B	0.48	0.830	0.625	0.245	0.314	0.142	0.076	0.504	0.109	0.306
IDEFICS-9B	0.24	0.781	0.621	0.229	0.296	0.128	0.070	0.482	0.099	0.290
LLaVA-1.5	1.18	0.720	0.617	0.218	0.295	0.108	0.059	0.462	0.083	0.273
**Small VLMs (LoRA Adapter)**										
MoonDream2	3.85	0.757	0.586	0.216	0.303	0.120	0.066	0.466	0.093	0.279
Qwen 2-VL	2.46	0.570	0.518	0.160	0.238	0.074	0.109	0.372	0.091	0.232
SmolVLM	8.34	0.414	0.536	0.136	0.252	0.016	0.060	0.362	0.038	0.200
Qwen-2.5	1.87	0.449	0.453	0.124	0.256	0.048	0.064	0.320	0.056	0.188
**Baselines (Full Finetune)**										
VisionGPT2	All	0.389	0.546	0.118	0.247	0.022	0.035	0.325	0.028	0.177
CNN-Transformer	All	0.399	0.414	0.044	0.277	0.018	0.030	0.284	0.024	0.154

**Table 6:** Impact of modality-aware prompting on SVLM performance. Modality labels were predicted by a ResNet-50 classifier and added at inference time. Italics indicate calculated percentage changes between configurations, distinguishing derived values from directly measured metrics.

Model	Configuration	Similarity	BERTScore	ROUGE	BLEURT	UMLS F1	AlignScore	Relevance Avg	Factuality Avg	Overall
SmolVLM	Base	0.414	0.536	0.136	0.252	0.016	0.060	0.362	0.038	0.200
SmolVLM	+Modality	0.418	0.532	0.144	0.266	0.031	0.096	0.365	0.048	0.207
	*Change*	*+1.0%*	*−0.7%*	*+5.9%*	*+5.6%*	*+93.8%*	*+60.0%*	*+0.8%*	*+26.3%*	*+3.5%*
Qwen 2-VL	Base	0.570	0.518	0.160	0.238	0.074	0.109	0.372	0.091	0.232
Qwen 2-VL	+Modality	0.364	0.456	0.121	0.311	0.017	0.086	0.313	0.052	0.182
	*Change*	*−36.1%*	*−12.0%*	*−24.4%*	*+30.7%*	*−77.0%*	*−21.1%*	*−15.9%*	*−42.9%*	*−21.6%*
Qwen-2.5	Base	0.449	0.453	0.124	0.256	0.048	0.064	0.320	0.056	0.188
Qwen-2.5	+Modality	0.502	0.461	0.122	0.268	0.032	0.065	0.351	0.049	0.200
	*Change*	*+11.8%*	*+1.8%*	*−1.6%*	*+4.7%*	*−33.3%*	*+1.6%*	*+9.7%*	*−12.5%*	*+6.4%*

**Table 7:** Component analysis for the chest X-ray. Components are highlighted as follows: Image Modality, Pathological Finding, Anatomical Location, Laterality, Visual marker. Laterality and visual markers are caption elements that aid clinical interpretation but are not UMLS concepts.

Ground Truth	chest X-ray showing a right pleural effusion (green arrow)
LLaVA-Mistral-7B	Chest X-ray showing a right-sided pleural effusion (green arrow)
LLaVA-Vicuna-7B	Chest X-ray showing a large right-sided pleural effusion (green arrow)
LLaVA-1.5	Chest X-ray showing a large right-sided pleural effusion
IDEFICS-9B	Chest X-ray showing a large right-sided pleural effusion (green arrow)
MoonDream2	Chest X-ray showing a right-sided pleural effusion (arrow)
Qwen 2-VL	Chest X-ray of the patient shows an enlarged right-sided infiltrate in the right lower lobe (arrow)
SmolVLM	An arrow pointing to a mass in the patient’s chest on a chest x-ray
Qwen 2.5	CT scan of the left kidney showing a renal cystic lesion (arrow)
VisionGPT2	A chest X-ray showing a large right-sided pneumothorax
CNN-Transformer	Chest x ray in sagittal

**Table 8:** Component analysis for the CT scan. Components are highlighted as follows: Image Modality, Pathological Finding, Anatomical Location, Clinical Measurement. Clinical measurements are caption elements that aid clinical interpretation but are not UMLS concepts.

Ground Truth	CT pulmonary embolus study showing pericardial effusion measuring 19.27 mm in this plane
LLaVA-Mistral-7B	CT pulmonary embolus study showing pericardial effusion measuring 19.27 mm in this plane
LLaVA-Vicuna-7B	CT pulmonary angiogram showing pericardial effusion measuring 19.27 mm in this plane
LLaVA-1.5	CT pulmonary embolism showing a large right lower lobe pulmonary embolism
IDEFICS-9B	CT pulmonary embolism study showing [truncated/incomplete]
MoonDream2	CT scan of the chest showing a large mass in the right upper lobe
Qwen 2-VL	Computed tomography scan showed a large right-sided mass in the right side
SmolVLM	A cross-section of a CT scan of the chest and abdomen, with a yellow square pointing to the 19.27 mm measurement
Qwen 2.5	Axial T2 magnetic resonance imaging of patient
VisionGPT2	PET-CT scan showing a large right hepatic cyst in the right hepatic lobe
CNN-Transformer	CT scan showing enlargement in liver lesion

## Data Availability

The Radiology Objects in Context version 2 (ROCOv2) dataset used in this study was obtained through ImageCLEFmed Caption Prediction Task, accessed on 5 March 2025, at https://www.imageclef.org. The dataset comprises radiological images and captions derived from PubMed Central open-access publications. Access requires registration with ImageCLEF.

## Appendix A. Analysis of Relevance–Factuality Relationship

Figure 9 reveals clear clustering patterns where LVLMs occupy the upper-right quadrant, baselines cluster in the lower-left, and SVLMs show intermediate performance. To quantify this observed pattern, we conducted an exploratory statistical analysis of the association between relevance and factuality metrics across the 10 evaluated models.

### Appendix A.1. Spearman’s Rank Correlation

Spearman’s rank correlation coefficient (ρ) was computed to measure the monotonic association between Relevance Scores and Factuality Scores [60]:

ρ=0.952,95%BootstrapConfidenceInterval:[0.688,1.000]

The confidence interval was estimated using bootstrap resampling with 10,000 iterations [61]. The observed correlation (ρ=0.952) indicates that within our model set, higher relevance scores consistently correspond to higher factuality scores. The bootstrap confidence interval reflects uncertainty due to the small sample size (*n* = 10), though, notably, even the lower bound (0.688) represents a strong positive association, supporting the clustering patterns visible in Figure 9.

### Appendix A.2. Regression Analysis

A linear regression line is shown in Figure 9 to illustrate the trend. The relationship appears approximately linear across the observed range, with $R^{2}=0.849$. While formal regression inference is not appropriate given the sample size, the fitted line provides visual reference for the strong positive association between the two metric categories.

### Appendix A.3. Interpretation and Scope

This analysis demonstrates that within our evaluated model set, relevance and factuality metrics co-vary substantially. Models with higher relevance scores also tend to have higher factuality scores. This observed pattern exhibited the following characteristics:

- The two metric categories, while measuring distinct aspects of caption quality (linguistic similarity vs. medical accuracy), are not independent within our model set.
- Models that perform well on one dimension tend to also perform well on the other dimension.
- The relationship is monotonic and approximately linear across the performance range represented by current VLM architectures.

Important limitations apply to this analysis. The analysis is observational and does not establish causality. The 10 models represent specific architectural choices rather than random samples from a population. The wide confidence interval reflects limited statistical power, and generalizability to other model architectures or datasets requires further investigation. Nonetheless, the observed pattern provides useful insight into how these complementary evaluation dimensions relate within current VLMs for medical image captioning.

## Appendix B. Computational Infrastructure

This appendix provides comprehensive hardware and software specifications for all experiments conducted in this study. Table A1 details the computational resources, software frameworks, and model allocation across different machine configurations.

**Table A1:** Complete hardware and software specifications for model training and evaluation.

Specification	Large Vision-Language Models	Small Vision-Language Models and Baselines
**Hardware Configuration**		
Machine Type	a2-highgpu-2g	n1-highmem-16
GPU Configuration	2 × NVIDIA A100-40GB	2 × NVIDIA V100
vCPUs	24	16
System RAM	170 GB	104 GB
Boot Disk	1000 GB SSD	150 GB SSD
Data Disk	1000 GB SSD	1000 GB SSD
Cloud Platform	Google Cloud Platform	Google Cloud Platform
**Software Environment**		
Operating System	Linux (Ubuntu-based)	Linux (Ubuntu-based)
Python Version	3.12	3.12
Primary Framework	PyTorch 2.2.1	PyTorch 2.2.1 (SVLMs), TensorFlow 2.16 (Baselines)
CUDA Version	12.1	12.1
cuDNN Version	Compatible with CUDA 12.1	Compatible with CUDA 12.1
Key Libraries	Hugging Face Transformers, PEFT, Flash Attention 2	Transformers, PEFT, Keras (CNN-Transformer)
Mixed Precision	bfloat16	bfloat16 (SVLMs), FP16 (VisionGPT2), TF AMP (CNN-Transformer)
**Model Allocation**		
Models Trained	LLaVA-Mistral-7B, LLaVA-Vicuna-7B	MoonDream2, Qwen-2-VL, Qwen-2.5-3B
	LLaVA-1.5-7B, IDEFICS-9B-Instruct	SmolVLM-500M, VisionGPT2, CNN-Transformer
**Storage Configuration**		
Dataset Storage	Persistent SSD (Data Disk)	Persistent SSD (Data Disk)
Model Checkpoints	Persistent SSD (Data Disk)	Persistent SSD (Data Disk)
Temporary Files	Boot Disk	Boot Disk

The differentiated hardware allocation strategy was designed to optimize resource utilization while maintaining consistency within model categories for fair performance comparison. LVLMs with 7 B to 9 B parameters required high-memory GPU configurations (A100-40GB) to accommodate model weights, optimizer states, and gradient computations during training. SVLMs and baseline architectures, with substantially fewer parameters, were efficiently trained on V100 GPUs or single A100-80GB GPUs. All models utilized persistent SSD storage for dataset caching and checkpoint management, ensuring consistent I/O performance across training runs. Training scripts are publicly available [62].

## References

[1] Afshari Mirak S., Tirumani S.H., Ramaiya N., Mohamed I. (2025). The growing nationwide radiologist shortage: Current opportunities and ongoing challenges for international medical graduate radiologists. Radiology.

[2] Rawson J.V., Smetherman D., Rubin E. (2024). Short-term strategies for augmenting the national radiologist workforce. Am. J. Roentgenol..

[3] Achour N., Zapata T., Saleh Y., Pierscionek B., Azzopardi-Muscat N., Novillo-Ortiz D., Morgan C., Chaouali M. (2025). The role of AI in mitigating the impact of radiologist shortages: A systematised review. Health Technol..

[4] Beddiar D.R., Oussalah M., Seppänen T. (2023). Automatic captioning for medical imaging (MIC): A rapid review of literature. Artif. Intell. Rev..

[5] Li T., Wang J., Jin L. (2024). Enhancing Visual Information Extraction with Large Language Models Through Layout-Aware Instruction Tuning. Proceedings of the Chinese Conference on Pattern Recognition and Computer Vision (PRCV).

[6] Alayrac J.B., Donahue J., Luc P., Miech A., Barr I., Hasson Y., Lenc K., Mensch A., Millican K., Reynolds M. (2022). Flamingo: A visual language model for few-shot learning. Adv. Neural Inf. Process. Syst..

[7] Radford A., Kim J.W., Hallacy C., Ramesh A., Goh G., Agarwal S., Sastry G., Askell A., Mishkin P., Clark J. Learning transferable visual models from natural language supervision. Proceedings of the International Conference on Machine Learning, PmLR.

[8] Bannur S., Hyland S., Liu Q., Perez-Garcia F., Ilse M., Castro D.C., Boecking B., Sharma H., Bouzid K., Thieme A. Learning to exploit temporal structure for biomedical vision-language processing. Proceedings of the IEEE/CVF Conference on Computer Vision and Pattern Recognition.

[9] Moor M., Huang Q., Wu S., Yasunaga M., Dalmia Y., Leskovec J., Zakka C., Reis E.P., Rajpurkar P. Med-flamingo: A multimodal medical few-shot learner. Proceedings of the Machine Learning for Health (ML4H), PMLR.

[10] Wu C., Zhang X., Zhang Y., Hui H., Wang Y., Xie W. (2025). Towards generalist foundation model for radiology by leveraging web-scale 2d&3d medical data. Nat. Commun..

[11] Ryu J.S., Kang H., Chu Y., Yang S. (2025). Vision-language foundation models for medical imaging: A review of current practices and innovations. Biomed. Eng. Lett..

[12] Laurençon H., Saulnier L., Tronchon L., Bekman S., Singh A., Lozhkov A., Wang T., Karamcheti S., Rush A., Kiela D. (2023). Obelics: An open web-scale filtered dataset of interleaved image-text documents. Adv. Neural Inf. Process. Syst..

[13] Kurz C.F., Merzhevich T., Eskofier B.M., Kather J.N., Gmeiner B. (2025). Benchmarking vision-language models for diagnostics in emergency and critical care settings. npj Digit. Med..

[14] Hoque M., Hasan M.R., Emon M.I.S., Oluwafemi E.P.O., Rahman M.M., Khalifa F. Comparative Analysis of Fine-Tuned Multimodal Models in Radiology Image Captioning. Proceedings of the 2025 IEEE 4th International Conference on Computing and Machine Intelligence (ICMI).

[15] Fang M., Wang Z., Pan S., Feng X., Zhao Y., Hou D., Wu L., Xie X., Zhang X.Y., Tian J. (2025). Large models in medical imaging: Advances and prospects. Chin. Med. J..

[16] Danish S., Sadeghi-Niaraki A., Khan S.U., Dang L.M., Tightiz L., Moon H. (2025). A comprehensive survey of Vision-Language Models: Pretrained models, fine-tuning, prompt engineering, adapters, and benchmark datasets. Inf. Fusion.

[17] Mei X., Shun J., Chao K. (2024). Efficient Fine-Tuning with Low-Rank Adaptation for Large-Scale AI Models. https://papers.ssrn.com/sol3/papers.cfm?abstract_id=5173161.

[18] Karmanov A., Guan D., Lu S., El Saddik A., Xing E. Efficient test-time adaptation of vision-language models. Proceedings of the IEEE/CVF Conference on Computer Vision and Pattern Recognition.

[19] Hu E.J., Shen Y., Wallis P., Allen-Zhu Z., Li Y., Wang S., Wang L., Chen W. (2022). Lora: Low-rank adaptation of large language models. ICLR.

[20] Zanella M., Ben Ayed I. Low-rank few-shot adaptation of vision-language models. Proceedings of the IEEE/CVF Conference on Computer Vision and Pattern Recognition.

[21] Hartsock I., Rasool G. (2024). Vision-language models for medical report generation and visual question answering: A review. Front. Artif. Intell..

[22] Hofmeister J., Garin N., Montet X., Scheffler M., Platon A., Poletti P.A., Stirnemann J., Debray M.P., Claessens Y.E., Duval X. (2024). Validating the accuracy of deep learning for the diagnosis of pneumonia on chest x-ray against a robust multimodal reference diagnosis: A post hoc analysis of two prospective studies. Eur. Radiol. Exp..

[23] Rückert J., Bloch L., Brüngel R., Idrissi-Yaghir A., Schäfer H., Schmidt C.S., Koitka S., Pelka O., Abacha A.B., G. Seco de Herrera A. (2024). Rocov2: Radiology objects in context version 2, an updated multimodal image dataset. Sci. Data.

[24] Bodenreider O. (2004). The unified medical language system (UMLS): Integrating biomedical terminology. Nucleic Acids Res..

[25] He K., Zhang X., Ren S., Sun J. Deep residual learning for image recognition. Proceedings of the IEEE Conference on Computer Vision and Pattern Recognition.

[26] Hasan M.R. (2024). Transformer and Convolutional Neural Network Based Hybrid Approaches in Medical Image Classification, Caption Generation, and Retrieval Processes. Master’s Thesis.

[27] Nam Y., Kim D.Y., Kyung S., Seo J., Song J.M., Kwon J., Kim J., Jo W., Park H., Sung J. (2025). Multimodal Large Language Models in Medical Imaging: Current State and Future Directions. Korean J. Radiol..

[28] Van M.H., Verma P., Wu X. On large visual language models for medical imaging analysis: An empirical study. Proceedings of the 2024 IEEE/ACM Conference on Connected Health: Applications, Systems and Engineering Technologies (CHASE).

[29] Liu H., Li C., Li Y., Lee Y.J. Improved baselines with visual instruction tuning. Proceedings of the IEEE/CVF Conference on Computer Vision and Pattern Recognition.

[30] Brown T., Mann B., Ryder N., Subbiah M., Kaplan J.D., Dhariwal P., Neelakantan A., Shyam P., Sastry G., Askell A. (2020). Language models are few-shot learners. Adv. Neural Inf. Process. Syst..

[31] Bai J., Bai S., Chu Y., Cui Z., Dang K., Deng X., Fan Y., Ge W., Han Y., Huang F. (2023). Qwen technical report. arXiv.

[32] Bai S., Chen K., Liu X., Wang J., Ge W., Song S., Dang K., Wang P., Wang S., Tang J. (2025). Qwen2. 5-vl technical report. arXiv.

[33] Li Y., Bubeck S., Eldan R., Del Giorno A., Gunasekar S., Lee Y.T. (2023). Textbooks Are All You Need II: **phi-1.5** technical report. arXiv.

[34] Marafioti A., Zohar O., Farré M., Noyan M., Bakouch E., Cuenca P., Zakka C., Allal L.B., Lozhkov A., Tazi N. (2025). Smolvlm: Redefining small and efficient multimodal models. arXiv.

[35] Dosovitskiy A., Beyer L., Kolesnikov A., Weissenborn D., Zhai X., Unterthiner T., Dehghani M., Minderer M., Heigold G., Gelly S. An Image is Worth 16x16 Words: Transformers for Image Recognition at Scale. Proceedings of the International Conference on Learning Representations.

[36] Radford A., Wu J., Child R., Luan D., Amodei D., Sutskever I. (2019). Language models are unsupervised multitask learners. OpenAI Blog.

[37] Shinde G., Ravi A., Dey E., Sakib S., Rampure M., Roy N. (2025). A Survey on Efficient Vision-Language Models. Wiley Interdiscip. Rev. Data Min. Knowl. Discov..

[38] Jin F., Zhang J., Zong C. Parameter-efficient tuning for large language model without calculating its gradients. Proceedings of the 2023 Conference on Empirical Methods in Natural Language Processing.

[39] Li M., Jiang Y., Zhang Y., Zhu H. (2023). Medical image analysis using deep learning algorithms. Front. Public Health.

[40] Liu H., Tam D., Muqeeth M., Mohta J., Huang T., Bansal M., Raffel C.A. (2022). Few-shot parameter-efficient fine-tuning is better and cheaper than in-context learning. Adv. Neural Inf. Process. Syst..

[41] Al-Kababji A., Bensaali F., Dakua S.P. (2022). Scheduling techniques for liver segmentation: Reducelronplateau vs onecyclelr. Proceedings of the International Conference on Intelligent Systems and Pattern Recognition.

[42] Smith L.N. Cyclical learning rates for training neural networks. Proceedings of the 2017 IEEE Winter Conference on Applications of Computer Vision (WACV).

[43] Zhang J., Huang L., Tan H., Zheng Z., Guo H. A scalable BFloat16 dot-product architecture for deep learning. Proceedings of the Great Lakes Symposium on VLSI 2023.

[44] Dao T. FlashAttention-2: Faster Attention with Better Parallelism and Work Partitioning. Proceedings of the Twelfth International Conference on Learning Representations.

[45] Loshchilov I., Hutter F. Decoupled Weight Decay Regularization. Proceedings of the International Conference on Learning Representations.

[46] Cai L., Gao J., Zhao D. (2020). A review of the application of deep learning in medical image classification and segmentation. Ann. Transl. Med..

[47] Mao A., Mohri M., Zhong Y. Cross-entropy loss functions: Theoretical analysis and applications. Proceedings of the International Conference on Machine Learning, PmLR.

[48] Kingma D.P., Ba J. (2014). Adam: A Method for Stochastic Optimization. arXiv.

[49] Damm H., Pakull T.M., Becker H., Bracke B., Eryilmaz B., Bloch L., Brüngel R., Schmidt C.S., Rückert J., Pelka O. Overview of ImageCLEFmedical 2025–medical concept detection and interpretable caption generation. Proceedings of the CLEF.

[50] Codella N.C., Jin Y., Jain S., Gu Y., Lee H.H., Abacha A.B., Santamaria-Pang A., Guyman W., Sangani N., Zhang S. (2024). Medimageinsight: An open-source embedding model for general domain medical imaging. arXiv.

[51] Zhang T., Kishore V., Wu F., Weinberger K.Q., Artzi Y. BERTScore: Evaluating Text Generation with BERT. Proceedings of the 8th International Conference on Learning Representations, ICLR 2020.

[52] He P., Liu X., Gao J., Chen W. DeBERTa: Decoding-enhanced BERT with Disentangled Attention. Proceedings of the International Conference on Learning Representations.

[53] Lin C.Y. Rouge: A package for automatic evaluation of summaries. Proceedings of the Text Summarization Branches Out.

[54] Sellam T., Das D., Parikh A. BLEURT: Learning robust metrics for text generation. Proceedings of the 58th Annual Meeting of the Association for Computational Linguistics.

[55] Kraljevic Z., Searle T., Shek A., Roguski L., Noor K., Bean D., Mascio A., Zhu L., Folarin A.A., Roberts A. (2021). Multi-domain clinical natural language processing with MedCAT: The medical concept annotation toolkit. Artif. Intell. Med..

[56] Yim W.w., Fu Y., Ben Abacha A., Snider N., Lin T., Yetisgen M. (2023). Aci-bench: A novel ambient clinical intelligence dataset for benchmarking automatic visit note generation. Sci. Data.

[57] Liu Y., Ott M., Goyal N., Du J., Joshi M., Chen D., Levy O., Lewis M., Zettlemoyer L., Stoyanov V. (2019). RoBERTa: A Robustly Optimized BERT Pretraining Approach. arXiv.

[58] Du R., Liu C., Yu S., Yang P., Tian D., Sun E., Xiong J. (2025). Mixture of Prompts Learning for Vision-Language Models. Front. Artif. Intell..

[59] Xie Q., Chen Q., Chen A., Peng C., Hu Y., Lin F., Peng X., Huang J., Zhang J., Keloth V. (2024). Me-llama: Foundation large language models for medical applications. Res. Sq..

[60] Puth M.T., Neuhäuser M., Ruxton G.D. (2015). Effective use of Spearman’s and Kendall’s correlation coefficients for association between two measured traits. Anim. Behav..

[61] Eden S.K., Li C., Shepherd B.E. (2022). Nonparametric estimation of Spearman’s rank correlation with bivariate survival data. Biometrics.

[62] Hoque M., Hasan M.R. (2024). Medical Image Interpretation with Large Multimodal Models: Caption Prediction Code Repository. https://github.com/HoqueMahmudul/Medical-Image-Interpretation-with-Large-Multimodal-Models/tree/main/Caption%20Prediction.

